# Evaluation of expression of genes associated with post-thrombotic syndrome

**DOI:** 10.1590/1677-5449.202401022

**Published:** 2025-05-30

**Authors:** Ricardo André Viana Barros, Erika Mota Herenio, Mariana Rocha Maximiano, Julia Hellena Mendes Ribeiro, Octávio Luiz Franco, Robert Edward Pogue

**Affiliations:** 1 Hospital de Base do Distrito Federal – HBDF, Brasília, DF, Brasil.; 2 Universidade Católica de Brasília – UCB, Brasília, DF, Brasil.; 3 Universidade de Brasília – UnB, Brasília, DF, Brasil.; 4 Universidade Católica Dom Bosco – UCDB, Campo Grande, MS, Brasil.; 5 University of Edinburgh, Edinburgh, United Kingdom.; 6 Newcastle University, Newcastle-upon-Tyne, United Kingdom.

**Keywords:** post-thrombotic syndrome, gene expression, coagulation factor XIII, myeloperoxidase, vascular endothelial growth factor receptor-3, síndrome pós-trombótica, expressão gênica, fator de coagulação XIII, mieloperoxidase, receptor-3 do fator de crescimento endotelial vascular

## Abstract

Prediction of the development of post-thrombotic syndrome (PTS) among patients with deep venous thrombosis (DVT) is currently based on clinical characteristics alone; no reliable biomarkers are available. Coagulation Factor XIII A chain (F13A1) of the clotting cascade stabilizes the thrombus; myeloperoxidase (MPO) interacts with the endothelium; and Fm**s-**related tyrosine kinase 4 (FLT4), also known as Vascular Endothelial Growth Factor Receptor-3, encodes a vascular endothelium-derived growth factor receptor that participates in angiogenesis. In this study, MPO, FLT4, and F13A1 gene expression was evaluated to identify novel biomarkers of PTS. This study evaluated nine patients allocated to three different groups. The control group included three healthy patients (group I); the second group included three patients with DVT without PTS (group II); and the third group included three patients with PTS (group III). Expression of MPO, FLT4, and F13A1 was evaluated in all three groups. A decrease in FLT4 expression was observed in group II (ΔCt -2.71; gene expression 0.03, p=0.11) and a significant decrease was observed in group III (ΔCt -2.44; gene expression 0.01, p=0.05). A nonsignificant difference in MPO gene expression was found among the three groups. There was a notable and progressive increase in F13A1 expression in group III (ΔCt 6.54; gene expression 3.5, p=0.02). Despite the low sampling rate in the present study, the decreased FLT4 expression and increased of F13A1 expression may represent biomarkers of PTS in group III.

## Resumo

A previsão do desenvolvimento da síndrome pós-trombótica (SPT) em pacientes com trombose venosa profunda (TVP) baseia-se, atualmente, apenas em características clínicas, pois biomarcadores confiáveis não estão disponíveis. A cadeia alfa do fator XIII (F13A1) da cascata de coagulação estabiliza o trombo; a mieloperoxidase (MPO) interage com o endotélio; e a tirosina quinase 4 (FLT4), também conhecida como receptor-3 do fator de crescimento endotelial vascular, é um receptor do fator de crescimento derivado do endotélio vascular envolvido na angiogênese. Avaliar a expressão gênica de MPO, FLT4 e F13A1 para identificar novos biomarcadores de SPT. Este estudo incluiu nove pacientes estratificados em três grupos diferentes. O grupo-controle incluiu três pacientes saudáveis (grupo I); o segundo grupo incluiu três pacientes com TVP sem SPT (grupo II); e o terceiro grupo incluiu três pacientes com SPT (grupo III). A expressão gênica de MPO, FLT4 e F13A1 foi avaliada nos três grupos. Observou-se uma redução significativa na expressão de FLT4 (ΔCt -2,71; expressão gênica 0,03, p = 0,11 no grupo II; ΔCt -2,44; expressão gênica 0,01, p = 0,05 no grupo III) e uma diferença não significativa na expressão do gene MPO entre os grupos. No entanto, houve um aumento progressivo notável na expressão de F13A1 no grupo III (ΔCt 6,54; expressão gênica 3,5, p = 0,02). Apesar da baixa taxa de amostragem no presente estudo, a diminuição da expressão de FLT4 e o aumento da expressão de F13A1 podem representar biomarcadores de SPT no grupo III.

## INTRODUCTION

Deep venous thrombosis (DVT) occurs when blood in a particular vein transitions from a liquid to a solid state (clot formation), creating an obstacle to venous return and causing several signs and symptoms including swelling, pain, and changes in skin color. Veins in the lower limbs constitute the most common sites of formation of these clots; however, they can appear in any body segment. Etiology is based on genetic and environmental factors and there may be a tendency to form thromboses (thrombophilia).^1^ Between 30 and 50% of patients with DVT develop a complication known as post-thrombotic syndrome (PTS), whose pathophysiology is linked to either persistence of the thrombus (totally or partially) or to the lesion via the inflammatory cascade in the venous walls. PTS causes definitive effects, such as recurrent edema, pain, tiredness, change in skin color of the legs, and venous ulcers, with a marked decrease in quality of life.^2^ The economic impact of PTS was exemplified in a Canadian study that calculated approximately 50% higher patient spending on DVT complicated by PTS than in cases without PTS.^3^ Several studies have screened for a reliable predictor of PTS based on epidemiological profile data or biomarkers; however, a consensus has not yet been reached.^4,5^ Myeloperoxidase (MPO), an enzyme found in increased levels in cardiovascular diseases, including DVT, aids in the production of free radicals by neutrophils and monocytes during phagocytosis and by producing highly reactive oxidative species involved in endothelial dysfunction.^6^ Recently, meshes of DNA, histones, and proteases secreted by neutrophils, called neutrophil extracellular traps (NETs), have been described, functioning as a defensive element against external pathogens and also contributing to thrombosis.^7^ MPO is a marker of the presence of NETs owing to its intertwined presence in these DNA and histone meshes, especially in patients with thrombosis and infections.^8^ In animal models, suppression of NETs in thrombi also reduces the incidence of thrombus remaining for longer periods than seen in the presence of NETs.^9^ Endothelium-derived growth factor receptor type 3 (VEGFR3) is encoded by the FLT4 gene and is expressed in cell membranes involved in endothelial venous and lymphatic angiogenesis.^10^ This receptor signals via phosphoinositide 3-kinase/mitogen-activated protein kinase, protein kinase B, extracellular signal-regulated kinase 1/2, and c-Jun N-terminal kinase pathways. Recent studies have reported presence of lymph angiogenesis in perivascular tissues with increased expression of VEGF-C and its preferential receptors (VEGFR3) in the presence of arterial or venous occlusions.^11,12^ Factor XIII (FXIII) is a tetrameric protein formed by aggregation of two A subunits and two B subunits encoded by the F13A1 and F13B genes, respectively. The F13A1 gene is located at locus 6-p25-p24 and the F13B gene is located at locus 1-q31-q32. The A subunits form a tetramer enzyme, which is activated by thrombin and binds with calcium, activating a transglutaminase 1-like enzymatic function to produce fibrin cross-links. This stabilizes the thrombus and prolongs its life before dissolution by natural fibrinolytics (plasmin).^13^ In this study, we analyzed expression of MPO, FLT4, and F13A1 in patients with PTS and quantitatively compared expression between patients with and without PTS. An increase in gene expression of F13A1 followed by decreased expression of FLT4 was found in patients with PTS compared to expression in the control group and in individuals with DVT but without PTS. There are no similar studies seeking to compare the specific gene expression of these mRNA and correlating it with PTS. The lack of biomarkers for PTS is a reality and discovery of new ones could change the prognosis of DVT. Our study is initial exploratory research on gene expression analysis in post thrombotic syndrome.

## METHODS

### Subject selection and sample collection

The study was evaluated and approved by the Human Research Ethics Committee at the main center, the Catholic University of Brasilia, and at the co-participant Base Hospital of the Federal District (Opinion Number: 4825370, protocol numbers CAAE 47628721.2.0000.0029 and CAAE 47628721.2.3001.8153, respectively), and participants provided informed consent. This was a cross-sectional study with three groups of three patients each. Participants were treated at the vascular surgery service at the Base Hospital of the Federal District on an outpatient basis. Villalta’s score was used to establish the diagnosis and severity of PTS, as recommended by the International Society on Thrombosis and Haemostasis; a score greater than 4 points establishes a diagnosis.^2^ The participants were assigned to three groups. The inclusion criteria for all groups were: participants of both sexes over 18 years of age. Group one (healthy controls) consisted of participants without comorbidities and without a history of DVT. Group two (thrombosis without PTS sequelae) included participants with a history of only one episode of DVT in the lower limbs for more than six months, diagnosed by vascular ultrasound. Group three (patients with PTS) included participants with a history of DVT showing absence of complete recanalization of the thrombus on ultrasound and presenting a Villalta score greater than five points and a confirmed diagnosis of PTS.

The exclusion criteria for all groups were defined as: current pregnancy or pregnancy during the DVT episode that caused PTS; history of thrombophilia; body mass index >30 kg/m^2^; presence of lymphedema of any etiology; presence of any infection; liver and/or chronic renal failure; patients with neoplasms (in the present or in the past); and patients with chronic venous insufficiency with varicose veins in the lower limbs diagnosed before DVT. Patients treated using unfractionated heparins or low molecular-weight heparins were excluded because heparins inhibit polymerase chain reaction (PCR).^14^ Blood samples were collected from participants in all groups between 12:00 and 13:00h. The Shapiro-Wilks normality test for small samples was used. The Mann-Whitney test was used for hypothesis testing. Correlation data between Villalta scores (independent variable) and expression of the three genes (MPO, FLT4, and F13A1) were analyzed, and a p-value smaller than 0.05 was considered significant. JASP 0.16.3 software^TM^ (JASP team 2022) was used to perform statistical analysis.

### RNA isolation and cDNA synthesis

RNA was isolated from 4 mL of peripheral blood using the TRIzol^TM^ reagent (Thermo Fisher Scientific^TM^, Asheville, NC, USA) according to the protocol GMB003rB:20090216NP-biorepository, Cleveland Clinic Lerner Research Institute. RNA quantification was performed using Nanodrop^TM^ 2000 and Qubit^TM^ spectrophotometer (Thermo Fisher Scientific^TM^). cDNA synthesis was performed using a High-Capacity cDNA Reverse Transcription^TM^ kit following the manufacturer’s instructions (Thermo Fisher Scientific^TM^). RT-qPCR analysis Reverse transcription-quantitative PCR (RT-qPCR) analysis was performed using SYBR^TM^ Green PCR Master Mix (Thermo Fisher Scientific^TM^). Each reaction contained 5 μL of Master Mix, 0.2 μM of each primer (forward and reverse), and 2 μL of Cdna (diluted 10-fold in water) in a final volume of 10 μL. RT-qPCR runs were performed in a QuantStudio 3^TM^ Real-Time PCR System (Applied Biosystems^TM^, Waltham, MA, USA) using a program with a hot start at 95 °C for 10 min, followed by 45 cycles of 95 °C for 5 s, 60 °C for 10 s, and 72 °C for 1 min. The melting curve was held after the end of amplification using the following steps: 95 °C for 15 s, 60 °C for 60 s, and a gradual increase of 0.3 °C up to 95 °C. Glyceraldehyde 3-phosphate dehydrogenase mRNA was used as a reference. The gene primer sequences are shown in Table 1.PCR efficiency and Ct values were obtained using Real-Time PCR Miner software.^15^ Relative expression (fold change) and statistics were evaluated using the relative expression software tool (REST 2009 v.2.0.13^TM^) to calculate expression values and for statistical validation.^16^ All experiments were performed using three biological replicates (each patient was considered a biological replicate) and three technical replicates.

**Table 1:** Gene primer features with sequence forward and reverse in first column and amplicon size in second column.

**Primers**	**Sequence**	**Amplicon size**
*F13A1 foward/reverse*	ATC CTG GGA CAA TAT CTA TGC/AAT GTG TTA AAG ACA CCA GC	141 pb
*FLT4 foward/reverse*	AGG TAT TAC AAC TGG GTG TCT/TC CTC AAA TGT CTT CAT CC	87 pb
*MPO foward/reverse*	CCA TGG AAC TCC TAT CCT ACT/TG ACT TGG ACA ACA CAT TC	183 pb

## RESULTS

As this is a preliminary study, we would need a sample of approximately 30 participants per group to obtain the value of the standard deviation of the gene expressions of the genes studied, which is an unknown value, and calculate the number of samples necessary for the inferential statistics tests. Despite this, the standard deviation of the mean values of the main difference in gene expression (F13A1, in our study) was 5.8. When using the sample calculation formula, n = (Z x standard deviation/standard error)^2^, where Z is the critical value for the desired confidence level (95%), the result would be approximately 5 participants, considering Z=1, 96 and an acceptable standard error of 5%.^17^

### Analysis of clinical characteristics

The sex split was 05 male and 04 female. Overall mean age was 36.4 years, ranging from 18 to 67 years; mean age in the control group was 32 years (18-50 years), mean age in group two was 36 years (35-55 years), and mean age in group three was 44 years (35-67 years). To facilitate data recording, lower limb segments were classified into the first segment, comprising the common and external iliac veins and the common, deep and femoral veins; second segment, comprising the popliteal vein; and third segment, comprising gastrocnemius, sural, fibular and tibial veins, which were all analyzed via Doppler ultrasound on the day of blood collection. The most frequently affected vein was the femoral vein in all groups; however, in group three (patients with PTS), there was a higher number of affected venous segments (mean 1.5 ± 0.57 versus mean 2.5 ± 0.57).The area of recanalization in group three was measured via vascular ultrasound in color mode and the average value was found to be 54.1% (95% CI, 0.002–0.723), with one case not showing recanalization. 

### Gene expression analysis

**Figure 1:**
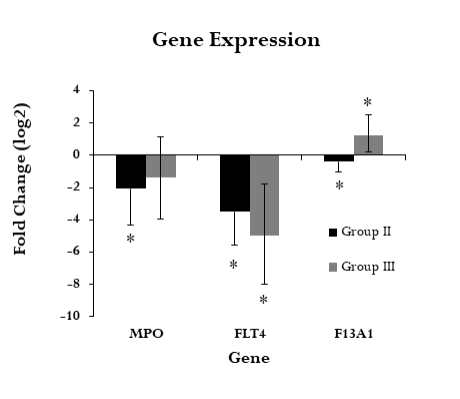
Relative expression of genes associated with PTS. Gene expression values were calculated using the relative expression software tool (REST) (ΔΔCt method) and normalized with the expression of the reference gene *GAPDH*. Each value represents the mean of three independent experiments with three technical replicates; mean ± standard error values (expressed in log_2_). (*) in bars indicate statistical significance (p ≤ 0.05). GAPDH, glyceraldehyde 3-phosphate dehydrogenase; MPO, myeloperoxidase; FLT4, Fms-related tyrosine kinase 4; F13A1, coagulation factor XIII A chain; PTS, post-thrombotic syndrome; Group II, patients with deep vein thrombosis but without PTS; Group III, patients with deep vein thrombosis and PTS.

**Figure 2:**
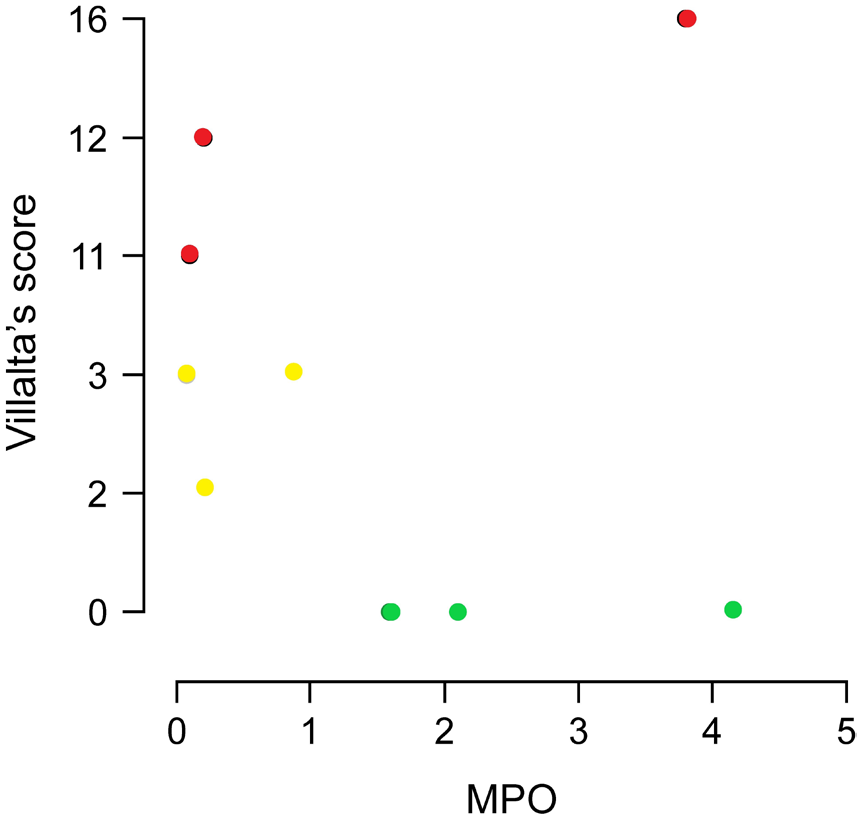
Comparison correlation of clinical score versus MPO expression in three groups (control in green, group 2 in yellow, and group 3 in red) calculated using the REST software (ΔΔCt method). MPO, myeloperoxidase; Group 2, patients with deep vein thrombosis but without post-thrombotic syndrome; Group 3, patients with deep vein thrombosis and post-thrombotic syndrome.

**Figure 3:**
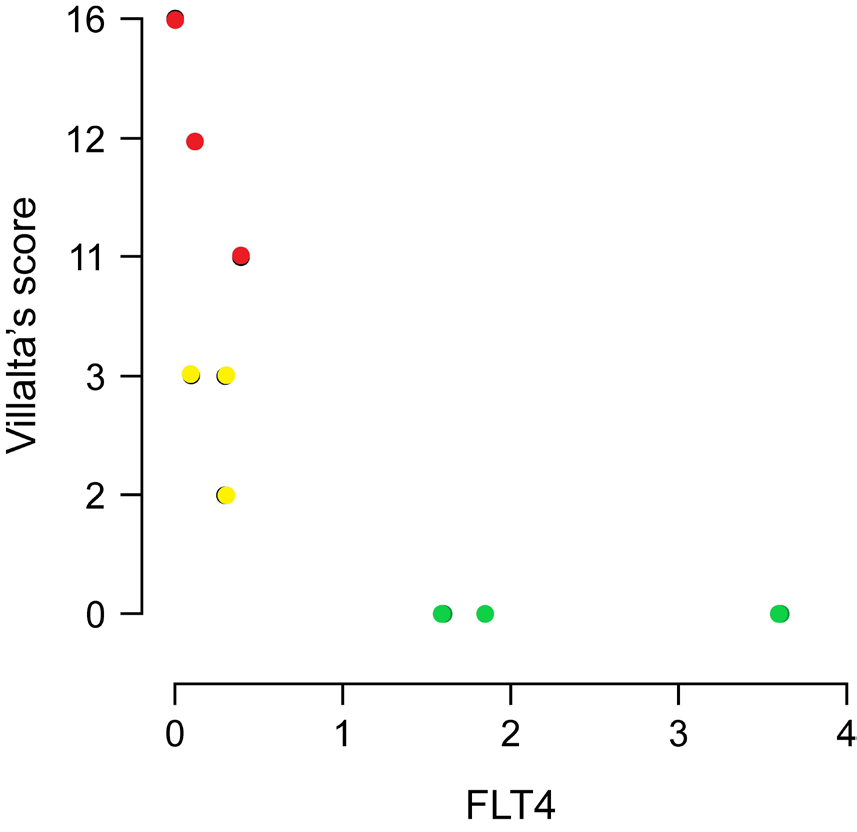
Comparison correlation of clinical score versus *FLT4* expression in three groups (control in green, group 2 in yellow, and group 3 in red) calculated using the REST software (ΔΔCt method). Group 2, patients with deep vein thrombosis but without post-thrombotic syndrome; Group 3, patients with deep vein thrombosis and post-thrombotic syndrome; FLT4, Fms-related tyrosine kinase 4.

**Figure 4:**
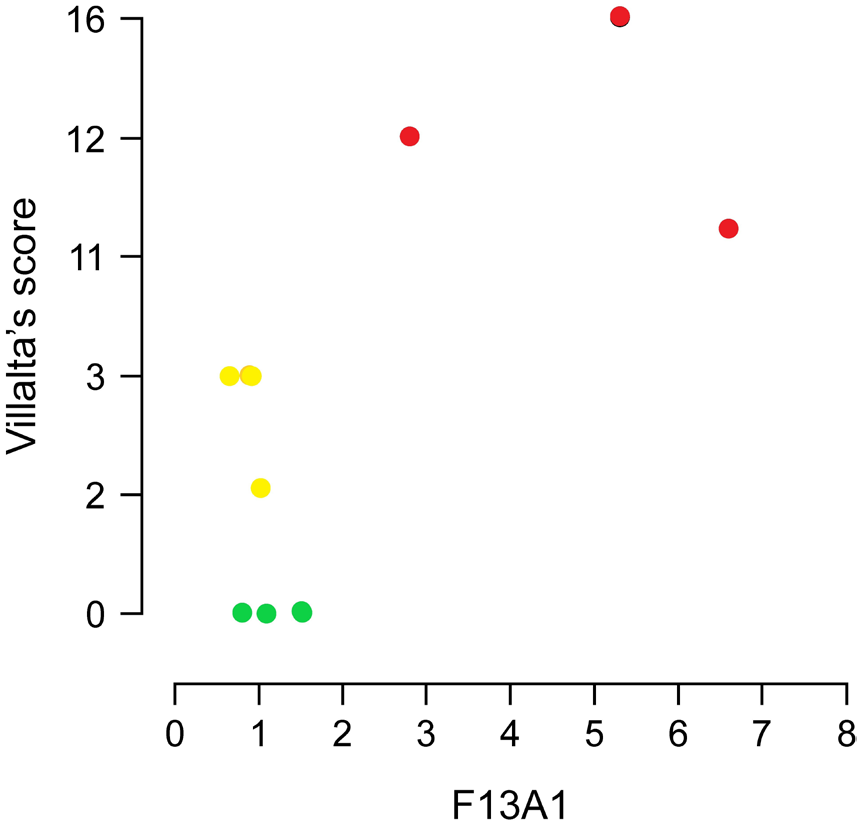
Comparison correlation of clinical score versus F13A1 expression in three groups (control in green, group 2 in yellow, and group 3 in red) calculated using the REST software (ΔΔCt method). F13A1, coagulation factor XIII A chain; Group 2, patients with deep vein thrombosis but without post-thrombotic syndrome; Group 3, patients with deep vein thrombosis and post-thrombotic syndrome.

Gene expression in groups two and three (Figure 1) was evaluated relative to expression in group one (healthy patients). MPO expression was decreased in group two participants (ΔCt -1.15; gene expression 0.08, p=0.34); there was no significant difference in the expression between group three and control group participants (ΔCt 1.52; gene expression 0.18, p=0.68). The FLT4 gene, which encodes the FLT4 protein, is involved in venous and lymphatic angiogenesis; FLT4 expression was decreased in groups two (ΔCt -2.71; gene expression 0.03, p=0.11) and three (ΔCt - 2.44; gene expression 0.01, p=0.05). F13A1 gene expression was considerably increased in group three (ΔCt 6.54; gene expression 3.5, p=0.02). In group two, the correlation between the signs and symptoms clinical score and gene expression was calculated using the relative expression software tool (ΔΔCt method). Patients in group two showed a negative correlation with MPO expression (p = 0.934; 95% CI: −0.682–0.646) (Figure 2) and FLT4 expression in the same group showed a negative correlation which was significant (p = 0.02; 95% CI: −0.910–0.073) (Figure 3). A slight decrease in F13A1 expression was observed in group two; while patients in group three showed a significant increase in F13A1 expression (p = 0.007; 95% CI, 0.335–0.960) (Figure 4).

## DISCUSSION

MPO is a marker of the presence of NETs and cellular remnants of platelet activating neutrophils, which are implicated in thrombotic processes.^18^ Formation of a thrombus and the inflammatory cascade involve biochemical pathways that intersect at different points and are indicated by many of their enzymes; studies have implicated MPO as an inhibitor of pro-coagulant activity and with a neutral role.^19,20^ The activity levels differ between antioxidant enzymes; an increase in superoxide dismutase activity and decrease in glutathione peroxidase activity has been reported in patients with DVT.^21^ In a comparative analysis of the transcriptome of leukocytes from patients with thrombosis with and without PTS, a difference in gene expression was found and sequenced in 12 different genes, messenger RNA and non-coding RNAs.^22^ Different biomarkers, based on endothelial dysfunction, increased inflammatory activity, and dysfunction in the coagulation cascade were tested and differences were found in serum dosages of CRP, ICAM-1, and E-selectin, which were increased in patients with PTS, while MMP9 and MCP-1 were decreased.^23^ We hypothesized that an increase in MPO gene expression may also contribute to PTS sequelae owing to the presence of thrombus remnants, thereby causing a possible capture of NETs consistently over time; however, we cannot verify this hypothesis with our results. Although MPO level is elevated in pro-coagulant states, such as in cancer and pregnancy,^24,25^ its gene expression may not necessarily be increased, since this enzyme may be obtained from degranulation of leukocytes and is present in NETs. FLT4 gene expression was decreased in participants in both groups two and three. Studies have reported indications of lymphatic proliferation associated with arterial obstructions;^26^ as seen in a study comparing peri-coronary adipose tissue from infarcted hearts with non-coronary perivascular adipose tissue (internal mammary artery used for revascularization). A considerable increase in FLT4 mRNA expression has been observed previously (0.13 vs 0.07, p = 0.02).^11^ In an experimental study of different phases of angiogenesis, the VEGF C/Flt4/ERK axis has been shown to promote cell cycle arrest and initiation of the sprouting phase. Accordingly, the decrease in FLT4 expression in the present study may be physiologically explained by the persistent formation of new vessels, especially in patients with PTS.^25^ This finding cannot be interpreted as a possible characteristic to compose a diagnostic panel since a larger number of study participants would be required to rule out type I statistical error. The increased activity of F13A1 in patients with DVT has already been described; however, studies had not previously evaluated its role in PTS. Several factors must be considered, such as the presence of variability between samples due to polymorphisms, as well as possible protection against thromboembolism in patients with a Val34Leu polymorphism in the F13A1 gene.^26^ Genotyping was not performed for the samples in the present study. The relationship between increased F13A1 activity and acute myocardial infarction in young patients was studied, and a possible risk factor for thrombosis was found.^27^ This corroborates the hypothesis suggested by our research in the sense that there is possibly higher activity of coagulation FXIII in more severe thrombosis as seen in enhanced F13A1 expression. An observational study of a cohort of 98 patients with coronavirus disease 2019 was recently published, in which a concentration of FXIII protein considerably below the normal limit was found. The authors attributed the decreased level to high consumption of coagulation factors; however, low synthesis or deficiency owing to immunological destruction was not completely ruled out.^28^ These observations suggest that the concentration of FXIII alone is not enough to elucidate its action, and studies on the expression of the genes involved, such as F13A1, can provide complementary data. Identification of new biomarkers will provide data for the construction of models for predicting and preventing PTS, as are already being built with technology using machine learning models aimed at PTS.^29^ However, our results cannot be interpreted as a biomarker diagnostic panel, as a greater number of participants in the study would be required to rule out type I statistical error. There are risks of bias inherent to cross sectional studies and risks of selection bias besides the inability to generalize the findings to different populations at this moment. To the best of our knowledge, this is the first time results related to increased expression of F13A1 in patients with PTS have been obtained, albeit these results were obtained using few samples. However, consistent and compatible statistical analyses were performed with small sample groups and normality testing. We aimed to find a relationship between increased expression of three different genes (MPO, FLT4, and F13A1); however, we found an increase in F13A1 gene expression and a decrease in FLT4 expression in patients with a Villalta score above 4 points (patients with PTS). Nevertheless, despite including homogeneous groups, our sample had a small number of participants per group. Our research provides a proof of concept that contributes to identification of biomarkers for PTS. Therefore, additional studies of the expression of MPO, FLT4, and F13A1 in patients with PTS are required to validate these results. Patients with DVT may benefit from being warned about the possibility of developing PTS right at the beginning of the condition, preventing the worsening of conditions with venous hypertension accompanied by edema and ulcers in the lower limbs. Our research will move on to a more relevant next step, increasing the number of samples from each group. New studies focusing on the gene expression of F13A1 and FLT4 and the dosage of the proteins themselves are necessary to ascertain their real usefulness as a biomarker to be employed at the population level. Further studies with larger sample sizes and randomized controlled clinical trial designs are needed to confirm the real potential of differential gene expression of the genes studied for use as biomarkers in PTS.
